# Gonad dose assessment in paediatric kidney nuclear medicine test using Monte Carlo simulation

**DOI:** 10.1093/jrr/rraa080

**Published:** 2020-09-15

**Authors:** Dong-Yeon Lee, Yong-Uk Kye, Hyo-Jin Kim, Jeung-Kee Kim, Yeong-Rok Kang

**Affiliations:** Dongnam Institute of Radiological & Medical Sciences, (46033) 40, Jwadong-gil, Jangan-eup, Gijang-gun, Busan, Republic of Korea; Dongnam Institute of Radiological & Medical Sciences, (46033) 40, Jwadong-gil, Jangan-eup, Gijang-gun, Busan, Republic of Korea; Dongnam Institute of Radiological & Medical Sciences, (46033) 40, Jwadong-gil, Jangan-eup, Gijang-gun, Busan, Republic of Korea; Dongnam Institute of Radiological & Medical Sciences, (46033) 40, Jwadong-gil, Jangan-eup, Gijang-gun, Busan, Republic of Korea; Dongnam Institute of Radiological & Medical Sciences, (46033) 40, Jwadong-gil, Jangan-eup, Gijang-gun, Busan, Republic of Korea

**Keywords:** gonad dose assessment, paediatric nuclear medicine test, Monte Carlo simulation, kidney function

## Abstract

In this study, we evaluated the effect of radiation dose on gonads during paediatric kidney nuclear medicine tests. Using Monte Carlo simulations, the distribution and effects of radiation were physically evaluated by displaying the distribution path of the source in the human body over time. In particular, the evaluation of doses in children, who are sensitive to radiation during nuclear medicine tests that use internal exposure among several types of medical exposures, was conducted to obtain data for the management of medical exposures. Our results indicated that under normal kidney function, the dose received by the target kidney was 0.430 mGy/mCi, which is ~6% higher than the dose suggested by the International Commission on Radiation Protection (ICRP). Furthermore, when kidney function was compromised, the dose estimated was 0.726 mGy/mCi, which is ~2% lower than the dose suggested by the ICRP. In the male and female gonads, namely the testicles and ovaries, the doses received were 0.359 mGy/mCi and 0.394 mGy/mCi, respectively, under normal kidney function. Similarly, under abnormal kidney function, the doses ranged from 0.187 to 0.353 mGy/mCi and 0.238 to 0.388 mGy/mCi in the male and female gonads, respectively.

## INTRODUCTION

In medical radiation, a nuclear medicine test is one in which radiopharmaceuticals are injected into the human body to generate an image of the radiation emitted from the body. This test is significantly different from the general X-ray test as it allows the examination of the anatomy of the human body as well as diagnosis and evaluation of physiological functions [1]. Therefore, this is an essential test in the medical field. However, as it requires radioactive isotopes to be injected into the human body, there can be internal exposures. Further, it is difficult to evaluate quantitative doses compared to general X-ray examination [2].

Accordingly, the International Commission on Radiation Protection (ICRP) has assessed and reported the radiation doses in patients exposed to radiopharmaceuticals [3, 4]. In addition, several national and international studies have examined the radiation doses that patients receive during a nuclear medicine test [5, 6]. However, because most of the previous studies evaluated the dose based on biokinetic data provided by the ICRP, the radiation dose distribution in the human body cannot be physically evaluated according to the actual movement of the radiation source.

**Fig. 1.**
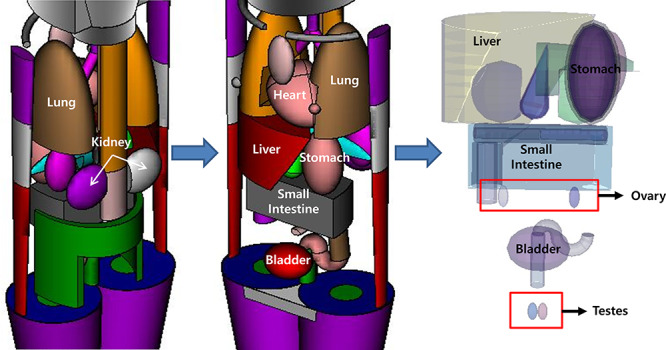
Paediatric human body modelled in 3D virtual space.

In addition, as medical radiation exposure offers benefits, no dose limit or regulation exists [7]. However, following the recent trends in controlling medical exposures, studies have been actively conducted to evaluate the radiation dose from medical exposures [8–10]. Among them, dose assessment for children is essential because their anatomical structure is different from adults in general and they are more sensitive to radiation.

In this study, we simulated the path of the radiopharmaceuticals in the human body according to physiological metabolism using a Monte Carlo program and evaluated the distribution of radiation physically. In particular, this study was conducted for highly sensitive children, and the data were acquired to manage medical exposure by evaluating the doses to the gonads, which are the most sensitive organs.

## MATERIALS AND METHODS

The organ dose evaluation in the case of internal exposure is limited by the half-life of the isotope injected into the human body and human physiological metabolism. In addition, it is impossible to evaluate the dose using a phantom for observing the internal exposure. This is because the phantom itself cannot simulate physiological metabolism. The purpose of this study was to evaluate the internal exposure dose by tracking the distribution of the source, which changes according to physiological metabolism, using the Monte Carlo simulation program (MCNP6.1.0) [11].

### Internal exposure dose evaluation system

Internal exposure is the exposure that occurs inside the human body when radioisotopes are ingested. The exposure dose can be assessed using the anatomy of the human body provided by the ICRP and biokinetic models of each organ and ingested nuclides. The said model was described in ICRP Publications 26, 30 and 66 [12–14], and the nuclides used as radiopharmaceuticals were summarised in ICRP Publications 80 and 106 [3,4] with the characteristics and patient doses for each nuclide specified. The nuclide used in the kidney function test, evaluated in this study, was ^99m^Tc-DTPA. According to the data provided by the ICRP, the radiation dose in a 5-year-old child was estimated to be 0.407 and 0.739 mGy/mCi when the dose in the kidneys was normal and abnormal, respectively. Further, for the gonads, the doses in the ovaries and testicles were estimated to be 0.407 mGy/mCi and 0.37 mGy/mCi, respectively, when the dose in the kidneys was normal, and 0.518 mGy/mCi and 0.407 mGy/mCi, respectively, when the dose in the kidneys was abnormal. However, the data presented by the ICRP have three major limitations. First, the positions of kidneys and gonads were not considered in the evaluation. Second, it was not specified whether the left or right kidney suffered from any abnormality. Finally, the degree of degradation of he kidney function was not considered in the evaluation. These limit the scope of quantitative dose data analysis for medical exposures of patients in actual nuclear medicine tests.

Therefore, in this study, doses for both the left and right sides of the kidneys and gonads were quantitatively evaluated, and the reliability of the results was ensured by comparing them with the results presented by the ICRP. In addition, the doses on the left and right sides of the organs were evaluated by dividing the degree of degradation of kidney function into several stages, and the evaluation data were collected to construct a database for proper management of medical exposure.

### Paediatric mock exposure

Figure 1 shows paediatric phantoms for mock exposures simulated in a 3D virtual space developed in the University of Florida (UF), USA and designed according to ages, from foetus to adult. Moreover, the male and female gonads were distinctly represented. In the case of the MIRD mock exposure phantom, which was manufactured for evaluating the internal dose, the organs were classified as soft tissue, lung and bone. In contrast, UF-revised exposure was expressed by segmenting 95 organs [15]. In addition, each organ, up to the mucosal layer, was included to accurately represent the actual organs, and anatomical structures and components of human organs were simulated based on ICRP89 [16] and ICRU46 [17]. The mock phantom used in this study was that of a 5-year-old child. The composition, density and volume of the major organs evaluated in this study are given in Table 1.

**Table 1:** Composition and percentage (%) of paediatric organs and density (g/cc) and volume (cc) of each organ

Organ								
	Lung	Heart	Stomach	Liver	Colon	Small intestine	Testis	Ovary
H	10.3	10.4	10.6	10.3	10.6	10.6	10.6	10.5
C	10.5	13.9	11.5	18.6	11.5	11.5	9.9	9.3
N	3.1	2.9	2.2	2.8	2.2	2.2	2.0	2.4
O	74.9	71.8	75.1	67.1	75.1	75.1	76.6	76.8
Na	0.2	0.1	0.1	0.2	0.1	0.1	0.2	0.2
P	0.2	0.2	0.1	0.2	0.1	0.1	0.1	0.2
S	0.3	0.2	0.1	0.3	0.1	0.1	0.2	0.2
Cl	0.3	0.2	0.2	0.2	0.2	0.2	0.2	0.2
K	0.1	0.3	0.1	0.3	0.1	0.1	0.2	0.2
Density	0.260	1.04	1.03	1.05	1.03	1.03	1.04	1.04
Volume	980	218	119.4	562	149.7	265	1.57	1.66

### Paediatric kidney test

The biokinetic model of the urinary system is related to the excretion of urine from the kidney to the bladder. Compared to the biokinetic model for other systems, this model yields a relatively simpler and clearer path. However, because the ability to filter urine varies depending on kidney function, it is necessary to carefully consider kidney function when evaluating internal exposure. Typically, kidney function is divided into normal and abnormal phases, depending on the glomerular filtration rate (GFR). The functional abnormality is considered high if the GFR is low. Table 2 lists the criteria for the GFR [18, 19]. The kidney test accounts for ~60% of nuclear tests for children, and most of them are dynamic tests that evaluate physiological functions rather than static tests of anatomical morphology [20]. The purpose of this study was to conduct a dynamic kidney test for evaluating the physiological function of the kidney. During this test, ^99m^Tc and DTPA were used as the medicine and marker, respectively. The basic characteristics of the isotope ^99m^Tc are given in Table 2 [21]. In general, dynamic testing is performed immediately after isotope injection to capture images in 1-min intervals to evaluate kidney function. Although variations depending on patient exist, the isotope is released from the kidney to the bladder in 20 min [22]. Therefore, the inspection time is ~20 min under normal conditions and increases up to 60 min in abnormal cases. Hence, in this study, the exposure dose in the gonads was evaluated, assuming a maximum test time of 60 min. In addition, the dose in the kidney was calculated and compared with the ICRP data [3, 4] to ensure the reliability of the calculation results. In the calculation, the ICRP assumed that the exposure time is 100 min under normal conditions and 1000 min under abnormal conditions. Therefore, for comparison, the doses in this study were calculated accordingly.

**Table 2:** National Kidney Foundation’s Kidney Disease Outcomes Quality Initiative (NKF-K/DOQI) stages of chronic kidney disease [18,19]

Stage	Description	GFR (mL/min/1.73 m^2^)
1	Kidney damage with normal or increased GFR	>90
2	Kidney damage with mild decrease in GFR	60–89
3	Moderate decrease in GFR	30–59
4	Severe decrease in GFR	15–29
5	Kidney failure	<15 or dialysis

**Table 3:** Detailed properties of ^99m^Tc

Nuclide	Radiation type	Half-life (h)	Energy (MeV)	Emission rate (%)
^99m^Tc	Gamma ray	6.01	0.0183	2.1
			0.0184	3.99
			0.1405	89.06

### Computational method

**Fig. 2.**
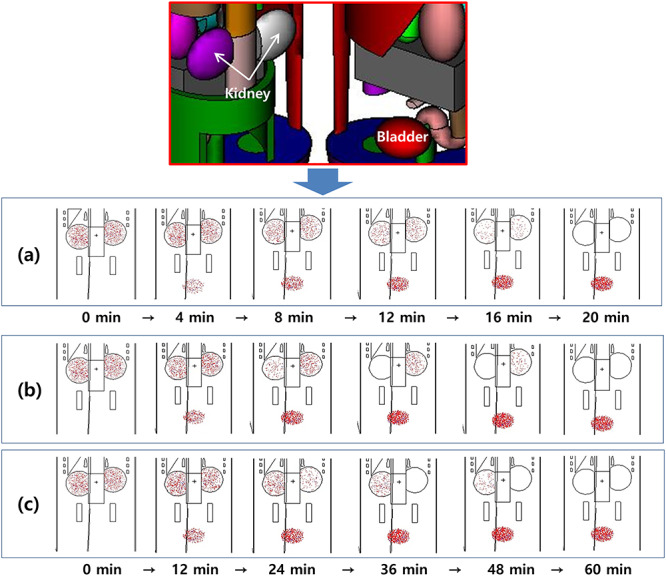
Simulation results for excretion of radiopharmaceuticals (^99m^Tc) from the kidney to the bladder using the MCNP code. The excretion process over time was simulated in 1-min intervals. (**a**) Normal kidney function, (**b**) degraded left kidney function, and (**c**) degraded right kidney function.

The physiological metabolism of the human body and radioactivity change due to the half-life of the isotope should be considered simultaneously to evaluate the internal dose owing to the isotope absorbed by the human body. In general, however, MCNP simulations cannot simulate the source location and radioactivity, which changes over time. To solve this problem, first, the source distribution due to physiological metabolism was simulated in 1-min intervals to calculate the dose. The degrees of kidney function was classified into 75, 50, 25 and 0%, where 100% indicates normal kidney function. Figure 2 shows the source diffusing over time.

**Fig. 3.**
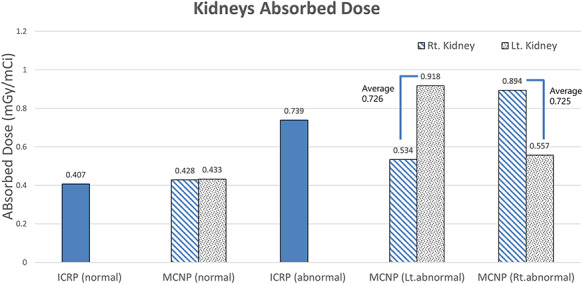
Kidney absorption doses presented by the ICRP and those calculated using the MCNP code for the case when left (Lt.) and right (Rt.) kidney function is abnormal.

**Fig. 4.**
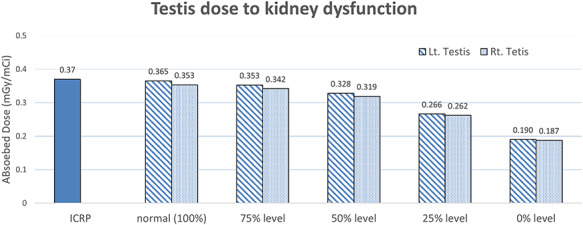
Testicle absorption doses presented by the ICRP and those calculated using the MCNP code. The evaluation was performed by classifying conditions into normal kidney function (100%) and abnormal kidney function (75, 50, 25, and 0%).

Second, as physiological metabolism occurs, the source changes its radioactivity according to its half-life. To account for this, the simulation results for each organ, obtained over time, were applied to equation (1) to calculate the dose in each organ.

(1) \begin{equation*} \mathrm{Organ}\ \mathrm{dose}={\int}_0^TA(t)\bullet{B}_0\bullet \exp \left(-\uplambda \bullet \mathrm{t}\right)\mathrm{dt} \end{equation*}

Here $A(t)$ is the organ dose calculated from the code representing the source flow in 1-min intervals, ${B}_0$ is the initial radioactivity of 1 mCi, λ is the decay constant of ^99m^Tc, *T* is the total time that the nuclide stays in the human body (100 min in normal conditions, 1000 min in abnormal conditions, and 60 min evaluation time for nuclear medicine nephrology according to the ICRP evaluation method), and *t* is the elapsed time. The reason for setting ${B}_0$ to 1 mCi is that the amount of radiopharmaceutical is different for each patient. In other words, if data are obtained by calculating the exposure dose based on 1 mCi, then the radiation dose can be predicted based on the amount of radiopharmaceuticals.

The simulation results were obtained using the tally option in the MCNP code. Among tally options, tally 6 was used to obtain the results in Mev/g, which were converted into mGy/mCi. In addition, to secure the reliability of the results calculated through the MCNP code, the number of simulation repetitions was set to 10^8^ to maintain the relative error within 3%.

## RESULTS

### Kidney absorption dose

To ensure the reliability of the results, the absorption dose in the kidney was evaluated and compared with that reported by the ICRP before calculating the doses in the gonads. Figure 3 graphically presents the kidney absorption doses provided by the ICRP and the results calculated using the MCNP code in this study.

**Fig. 5.**
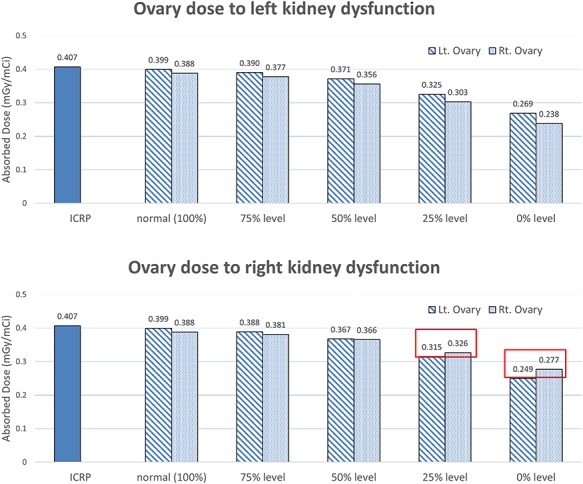
Ovarian absorption doses presented by the ICRP and those calculated using the MCNP code. The degree of degradation of the left (Lt.) and right (Rt.) kidney function is expressed in percentages, namely 75, 50, 25 and 0%, where 100% indicates normal kidney function.

Under normal kidney function conditions, the absorption doses calculated by the MCNP were 0.428 and 0.433 mGy/mCi in the right and left kidney, respectively. The value presented by the ICRP was 0.407 mGy/mCi, indicating that the calculated value is ~6% higher than the ICRP value. In the case of abnormal kidney function, the ICRP value was 0.739 mGy/mCi regardless of the side of the kidney. In this study, both left and right sides were evaluated in normal as well as abnormal conditions. The calculated values were 0.9 and 0.54 mGy/mCi for abnormal and normal kidney functions, respectively. In addition, the calculated average dose on both the left and right sides was 0.725 mGy/mCi, with the value being ~2% lower than the ICRP value.

### Testicular absorption dose

Figure 4 depicts the dose in the testicles, which correspond to the male genitalia. The doses calculated using the MCNP and data presented by the ICRP are summarised for comparison. In addition, the dose was calculated according to the degree of function degradation (75, 50, 25 and 0%), where 100% represents normal kidney function. The abnormal conditions are expressed for both the left and right kidneys. From the analysis, the calculated average dose for both the kidneys was 0.359 mGy/mCi under normal kidney function, and it is ~3% lower than the ICRP value. Further, the results indicate that the doses in the testicles decrease as kidney function degrades.

### Ovarian absorption dose

Figure 5 show the absorption dose of an ovary, which corresponds to the female genitalia. The calculated values of this study and the ICRP values are summarised for comparison. The dose change is represented by percentage values, 75, 50, 25 and 0%, indicating the increasing degree of degradation of kidney function.

In the first case, when kidney function was normal, the simulation results indicated 0.399 mGy/mCi for the left ovary and 0.388 mGy/mCi for the right ovary, and the average value was 0.393 mGy/mCi, which is ~3.5% lower than the ICRP value. Moreover, the dose was slightly higher in the left ovary.

Next, for the case when the right kidney function was abnormal, the ovarian dose tended to decrease as the function degraded, and the overall dose was slightly higher in the left ovary. In particular, the difference in the left and right ovary doses was remarkably reduced when right kidney function degraded to 50%, whereas the calculated right ovarian dose was higher than the calculated left ovarian dose when the function degraded to 25%.

Analysis was then performed for the case when the left kidney was abnormal. The ovarian dose tended to decrease as the function degraded, with the left ovary having a higher dose than the right ovary. In addition, the difference between the left and right ovary doses increases with the degree of degradation of the left kidney function, with the difference increasing to ~11% at 0.03 mGy/mCi when kidney function is 0%.

## DISCUSSION

The purpose of this study was to evaluate the internal exposure dose due to ^99m^TC-DTPA nuclide by simulating a paediatric human phantom in a 3D virtual space using the MCNP simulation code.

First, to ensure the reliability of the results in this study, the geometric structure of the simulated exposures was identified. Second, radiopharmaceuticals that diffuse according to physiological metabolism were represented in the simulation. Finally, the results calculated for the kidneys in this study were compared with previous studies. In this study, the MCNP visual programs (VisedX_24J [23], SABRINA [24] and SimpleGeo 4.3 [25]) were used to identify the geometry and constituents of the mock exposure phantom as shown in Fig. 1. Figure 2 shows the distribution of the source in the human body. From both the figures, it can be inferred that the anatomy of the human body and source are well reproduced. Finally, to ensure the reliability of the results, the absorption dose presented by the ICRP was compared with the results obtained in this study. The results indicate that the absorption dose was higher than the ICRP value by 6% when kidney function was normal and lower by 2% when kidney function was abnormal. The difference in the results may be due to the different evaluation methods, namely the differences in the geometry of the human body and the source setting. Further, the results can be trusted as the difference obtained is negligible.

Similarly, the calculated testicular dose in this study was different from the testicular dose presented by the ICRP by ~3%. This difference is likely owing to the dose evaluation method, and as the difference is within the margin of error, the results can be considered reliable. In addition, after the dose was analysed according to the degree of degradation of kidney function, it was found that testicular dose decreased as the function degraded. This is because the testicles are anatomically located under the bladder. As kidney function degrades, fewer sources are excreted into the bladder, and thus a lower testicular dose is obtained.

The simulation results obtained in this study show that the dose was generally determined to be high in the organs located on the left. This could be attributed to the anatomical location of the kidneys. The kidneys are located in the retroperitoneal space. In particular, the right kidney is located immediately below the liver, and the liver covers the upper part of the kidney. Thus, the right kidney is significantly affected by the liver, whereas the left kidney is less affected by the surrounding organs. Therefore, the dose to the organ located on the left was determined to be higher. The overall evaluation regarding the dose to the testicles and ovaries is as follows. The testicles, which are located under the bladder, do not affect the function of the left and right kidneys, and they are influenced by the amount of source that is excreted into the bladder by the kidney function. On the other hand, the ovaries are affected by the function of the left and right kidneys. The dose for the left ovary was determined to be high in the normal case, whereas the dose for the ovary located on the side with the abnormality increased when kidney function was abnormal.

Finally, the dose in the left ovary was slightly higher than that in the right ovary when kidney function was normal because of the anatomical location of the organs in the lower abdomen. In addition, the ovarian dose calculated in this study was different from that presented by the ICRP by ~3%. As described previously, this difference may be owing to the dose evaluation method, and because the difference is within the margin of error, the results are reliable. However, when the left and right ovarian doses were compared, the ovaries located on the side with the abnormal kidney function were observed to have a high dose. This phenomenon is attributed to the amount of radiopharmaceutical excreted by the bladder because the ovarian anatomical position is closer to the bladder than to the kidneys.

## CONCLUSION

In contrast to the calculation of the internal exposure dose through the biokinetic model proposed by the ICRP, this study analyses the radiation distribution due to the movement of the source through the simulated exposure phantom.

Further, as exposures generally experienced in the medical field were evaluated in this study, the data and the results can be utilized in medical exposure management. In particular, because the analysis was conducted on children with high radiation sensitivity, the acquired data should be useful.

Finally, there are some differences between the data obtained in this study and those presented by the ICRP. In this study, doses in each organ, including both kidneys, were evaluated according to the degree of degradation in kidney function. This analysis can be effective to systematically manage the exposure dose in patients according to their medical conditions.

## FUNDING

This work was supported by the Dongnam Institute of Radiological & Medical Sciences (DIRAMS) grant funded by the Korean government (MSIT) (No.50496–2020).

## CONFLICT OF INTEREST

None declared.
